# Platinum drug sensitivity and resistance in testicular germ cell tumors: two sides of the same coin

**DOI:** 10.20517/cdr.2020.24

**Published:** 2020-06-16

**Authors:** Pasquale Rescigno, Margaret Ottaviano, Giovannella Palmieri

**Affiliations:** ^1^Division of Clinical Studies, The Institute of Cancer Research and The Royal Marsden Hospital, London SM2 5NG, United Kingdom.; ^2^Division of Oncology, CRTR Rare Tumors Reference Center, AOU Federico II, Naples 80100, Italy.

Testicular germ cell tumors (TGCTs) represent the most common malignancies in young males but they also show the highest cure rate in solid tumors with more than 95% newly diagnosed TGCTs being ultimately cured^[1]^.

Singh *et al.*^[2]^ recently provided precious insight into the genomic aberrations underpinning the molecular characteristics of these tumors and a clear presentation of the mechanisms underlying sensitivity/resistance to platinum.

More importantly, the authors identified pre-target, on-target and post-target factors that can explain, the two sides of the same coin: the exquisite sensitivity of these cancers to platinum-based chemotherapy and at the same time, drug resistance when these factors are lacking.

Nucleotide excision repair (NER) is the main DNA repair system aimed at resolving platinum-induced adducts. RNA polymerase II stalls at the site of DNA damage, resulting in the recruitment of cockayne syndrome type A and type B proteins (CSA and CSB) (ERCC8 and ERCC6). Therefore, inactivating mutations or deletions in key elements of the NER system result in responsiveness to cisplatin (CDDP), suggesting that a lack of DNA damage recognition and repair determines damage accumulation, which ultimately increases apoptosis in these cells^[3]^. Indeed, single-strand breaks can result in double-strand breaks that are repaired, under normal conditions, by the homologous recombination system, of which BRCA2 and PARP are essential components. Defects in the homologous recombination system can also be responsible for sensitivity to CDDP^[4,5]^.

The role of the mismatch repair (MMR) system in TGCTs still remains controversial, but potentially, it can open new treatment venues not particularly investigated by the authors in their review^[2]^.

In the context of defective NER, the MMR system is believed to detect DNA adducts caused by CDDP, engage in their repair and ultimately fail, thus transmitting a pro-apoptotic signal^[6]^.

Therefore, genes encoding MMR components such as mutS homolog 2 (*MSH2*) and mutL homolog 1 (*MLH1*) are commonly mutated or downregulated in the context-acquired CDDP resistance in solid tumors^[7-9]^.

MMR deficiency has been proposed as a biomarker of response to immune-checkpoint inhibitors^[10,11]^. While a phase II trial with pembrolizumab in unselected platinum-refractory TGCTs showed the absence of any clinical benefit^[12]^ and despite MMR deficiency being uncommon in primary tumors, approximately 25% of platinum-resistant TGCTs displayed a phenotype characterized by microsatellite instability and lack of expression of MLH1 or MSH6^[13]^. Therefore, selecting platinum-refractory MMR-defective TGCTs could provide the right subset of patients likely to benefit from immune-checkpoint inhibition.

Lastly, Singh *et al*.^[2]^ discuss post-target and epigenetic causes of CDDP resistance in TGCTs, mainly focusing on p53/Mdm2 alterations.

High MDM2 expression is associated with more advanced stages (IIB, IIC and III) and increased risk of metastatic disease in TGCTs^[14,15]^, and it seems to be induced by the activation of the PI3K/AKT pathway^[2]^. These could represent new therapeutic targets, since MDM2 inhibitors^[16]^, as well as PI3K/AKT inhibitors^[17]^, are now under investigation as anticancer treatment in solid tumors. Moreover, several PI3K/AKT inhibitors have shown synergistic or additive effects with CDDP against different cancer cells *in vitro* and *in vivo* experiments^[18]^.

The authors also pointed out that epigenetic modification such as DNA methylation may be associated with CDDP resistance^[2]^. Accordingly, seminomas, which are generally considered more CDDP sensitive, are severely hypomethylated, while embryonal carcinomas, which show some platinum resistance, have an intermediate level of methylation, and teratomas, yolk sac tumors and choriocarcinomas have the highest level of DNA methylation^[2]^.

The relevance of epigenetics opens two relevant avenues for the treatment and the follow-up of TGCTs.

The epigenetic agents guadecitabine (a prodrug of decitabine, a DNMT1 inhibitor), animacroxam (an HDAC inhibitor), and JQ1 (an inhibitor of the BET family of bromodomain proteins) have shown promising effects on TGCTs in preclinical *in vivo* and *in vitro* studies^[19-21]^.

Moreover, small noncoding RNAs that are involved in the epigenetic regulation of gene expression, such as microRNAs 371a-3p, were found to be highly sensitive and specific biomarkers for TGCTs^[22]^.

In conclusion, the work from Singh *et al*.^[2]^ not only reviews the main mechanism of resistance to platinum-based therapies in TGCTs, but also sheds light upon new therapeutic approaches.

## Declarations

### Copyright

© The Author(s) 2020.
